# EHMTI-0238. Why narcissus was probably no migraineur: a study of visually induced analgesia

**DOI:** 10.1186/1129-2377-15-S1-E15

**Published:** 2014-09-18

**Authors:** S Sava, R Baschi, E Vecchio, V De Pasqua, J Schoenen, D Magis

**Affiliations:** 1 University Department of Neurology, Headache Research Unit, Liege, Belgium

## Background

Visually induced analgesia (VIA) corresponds to the decrease of pain perception when gazing at the stimulated body part, due to an interplay between the brain's pain and posterior visual networks. However VIA has never been studied in trigeminal areas. We know that migraine is associated with abnormal connectivity in visual areas.

## Aim

To study of trigeminal (face) and peripheral (wrist) VIA in healthy volunteers (HV) and migraine without aura patients (MO).

## Methods

Contact Heat Evoked Potentials (CHEPs), or thermonociceptive potentials, were obtained by heat stimulation (53°C) of the right forehead in 10 HS and 12 MO in interictal phase, able or not to gaze at their stimulated face reflected by a mirror. CHEPs were also obtained on the right wrist using the same protocol.

## Results

In HV, gazing at the face reflection decreased significantly CHEPs latency (N2) and amplitude (P1-N2 and N2-P2), and increased P1-N2 habituation. Conversely, the mirror considerably decreased trigeminal CHEPs habituation in MO. Pain perception assessed by visual analogue scale was not modified either by the mirror, or between groups. The mirror decreased CHEPs parameters in the wrist numerically but not significantly in both groups.

## Conclusions

The visually induced analgesia phenomenon using a mirror is demonstrated by a decrease of thermonociceptive potentials in HV in trigeminal and peripheral areas. In interictal migraineurs, forehead VIA is significantly impaired and gazing at the mirror strongly decreases the trigeminal habituation to thermal nociceptive stimulation. This study suggests a connectivity dysfunction between visual and trigeminal pain networks.

No conflict of interest.

